# Pleiotropic Genes for Pulmonary Function and Aging-Related Traits: The Long Life Family Study (LLFS)

**DOI:** 10.1093/geroni/igab046.534

**Published:** 2021-12-17

**Authors:** Mary Feitosa, Mary Wojczynski, Jason Anema, E Warwick Daw, Lihua Wang, Marianne Nygaard, Adam Santanasto, Michael Province

**Affiliations:** 1 Washington University School of Medicine in St. Louis, St. Louis, Missouri, United States; 2 Washington University School of Medicine, Washington University School of Medicine, Missouri, United States; 3 Washington University School of Medicine, St Louis, Missouri, United States; 4 Washington University in St. Louis, St. Louis, Missouri, United States; 5 Washington University School of Medicine, St. Louis, Missouri, United States; 6 Department of Public Health, University of Southern Denmark, Odense, Syddanmark, Denmark; 7 University of Pittsburgh, Pittsburgh, Pittsburgh, Pennsylvania, United States

## Abstract

Pulmonary function progressively declines with aging. Forced expiratory volume 1-second (FEV1) and forced vital capacity (FVC) are predictors of morbidity of cardiovascular diseases and all-cause mortality. Reduced pulmonary function has shown association with elevated chronic low-grade systemic inflammation and low muscle strength, and glycosylated hemoglobin (HbA1c). We evaluated whether FEV1 and FVC share common genetic factors with interleukin-6 (IL-6), high-sensitivity C-reactive protein (hsCRP), body mass index (BMI), handgrip strength, plasma glucose, and HbA1c employing correlated meta-analysis (CMA) in up to 3888 individuals (age range: 26-106). CMA tests whether combining genome-wide association (GWA) P-values from two or more traits enhances the ability to detect variants concomitantly influencing such traits. We considered a variant pleiotropic if the univariate GWA P≤1.0x10-02 and CMA P≤5.0x10-08. We identified pleiotropic loci for FEV1 (or FVC), IL-6 and hsCRP within CYCS, CALN1, TRIM9, AXIN2, MICAL3, and CMIP; FEV1 (or FVC) and BMI within CYP2U1, LINC00871-RPL10L-MDGA2, and LOC105372472; FEV1 (or FVC) and grip strength within XXYLT1 and MAL2; FEV1 and FPG within MAF; and FVC and HbA1c within HDHD3. Some loci were reported as GWAS suggestive (P<10-6) for pulmonary function. Additionally, the identified loci harbor genes with roles in pro-inflammatory cytokine production, immune and inflammatory pathway, T-helper signaling pathway, transforming growth factor-beta, and T-cell receptor-alpha enhancer. Our findings suggest that inflammation is a feature of reduced pulmonary function with IL-6, hsCRP, BMI, low muscle strength, glucose, and HbA1c. Pleiotropic genetic associations across these traits may explain part of the correlated genetic architecture between pulmonary function and aging-related traits.

